# β-Caryophyllene Mitigates Collagen Antibody Induced Arthritis (CAIA) in Mice Through a Cross-Talk between CB2 and PPAR-γ Receptors

**DOI:** 10.3390/biom9080326

**Published:** 2019-07-31

**Authors:** Natasha Irrera, Angela D’Ascola, Giovanni Pallio, Alessandra Bitto, Emanuela Mazzon, Federica Mannino, Violetta Squadrito, Vincenzo Arcoraci, Letteria Minutoli, Giuseppe Maurizio Campo, Angela Avenoso, Elisa Benedetta Bongiorno, Mario Vaccaro, Francesco Squadrito, Domenica Altavilla

**Affiliations:** 1 Department of Clinical and Experimental Medicine, University of Messina, 98125 Messina, Italy; 2 IRCCS Centro Neurolesi “Bonino-Pulejo”, 98124 Messina, Italy; 3 Department of Biomedical and Dental Sciences and Morphofunctional Imaging, University of Messina, 98125 Messina, Italy

**Keywords:** β-caryophyllene, CB2 receptors, PPAR-γ, CAIA, arthritis

## Abstract

β-caryophyllene (BCP) is a cannabinoid receptor 2 (CB2) agonist that tempers inflammation. An interaction between the CB2 receptor and peroxisome proliferator-activated receptor gamma (PPAR-γ) has been suggested and PPAR-γ activation exerts anti-arthritic effects. The aim of this study was to characterize the therapeutic activity of BCP and to investigate PPAR-γ involvement in a collagen antibody induced arthritis (CAIA) experimental model. CAIA was induced through intraperitoneal injection of a monoclonal antibody cocktail and lipopolysaccharide (LPS; 50 μg/100 μL/ip). CAIA animals were then randomized to orally receive either BCP (10 mg/kg/100 μL) or its vehicle (100 μL of corn oil). BCP significantly hampered the severity of the disease, reduced relevant pro-inflammatory cytokines, and increased the anti-inflammatory cytokine IL-13. BCP also decreased joint expression of matrix metalloproteinases 3 and 9. Arthritic joints showed increased COX2 and NF-ĸB mRNA expression and reduced expression of the PPARγ coactivator-1 alpha, PGC-1α, and PPAR-γ. These conditions were reverted following BCP treatment. Finally, BCP reduced NF-ĸB activation and increased PGC-1α and PPAR-γ expression in human articular chondrocytes stimulated with LPS. These effects were reverted by AM630, a CB2 receptor antagonist. These results suggest that BCP ameliorates arthritis through a cross-talk between CB2 and PPAR-γ.

## 1. Introduction

Rheumatoid arthritis (RA) affects 0.5–1.0% of the adult population worldwide and represents one of the main causes of disability and productive loss in countries with elevated income [1]. Autoimmunity and inflammation play key roles in the progression of the disease; moreover, synovia hypertrophy together with inflammation and tenderness of small joints represent the characteristic and relevant hallmarks of arthritis [2].

As far as the pathophysiology of this condition is concerned, it has been proposed that a genetic background together with an “environmental precondition” triggers an exaggerated immune reaction that induces the production of autoantibodies, including the rheumatoid factor and antibodies to citrullinated peptides [3,4]. This altered immune response boosts the activation and proliferation of inflammatory cells that infiltrate the tissues causing edema, cartilage damage, and granulation tissue production. This cascade of pathological events leads, at a later stage, to ankylosis and joint deformities.

The progression of the disease is characterized by a priming of inflammatory cells that release a plethora of pro-inflammatory cytokines, such as tumor necrosis factor (TNF-α), interleukin 1 beta (IL-1β), and IL-6 [5]. The acquisition of the inflammatory phenotype is induced by nuclear factor ĸB (NF-ĸΒ), a transcription factor that translocates upon activation to the nucleus and engages genes that codify for inflammatory proteins. In addition, several matrix metalloproteinases (MMPs) are released, concurring to empower an inflammatory reaction that causes joint destruction [6].

Several treatments have been proposed for RA including non-steroidal anti-inflammatory agents, disease-modifying medicines, and biological drugs to counteract the immune and inflammatory pathological cascade. All these therapeutic approaches have been shown to be effective in the management of rheumatoid arthritis, even if they are expensive and their chronic use in clinical practice is hampered by the occurrence of mild-to-moderate side effects [7,8,9,10].

Beta-caryophyllene (β-caryophyllene, BCP) is a biciclic sesquiterpene extracted from copaiba (Copaifera spp) and marijuana/hemp (Cannabis spp) that has received approval by the Food Drug Adminstration (FDA) because of its intriguing therapeutic potential. This plant-derived product has been used in traditional medicine because of its anti-inflammatory and analgesic effects [11]. β-caryophyllene binds the cannabinoid CB2 receptors which are primarily expressed in the immune and immune-derived cells [12]. Type 1 and type 2 cannabinoid receptors (CB1R and CB2R, respectively) are two subtypes of G-protein coupled receptors of the endocannabinoid system. CB2R is involved in the modulation of inflammatory response not only at the immune system level; in fact, it has been demonstrated that CB2Rs are also expressed in other areas such as in brain regions [13]. Moreover, the corticolimbic endocannabinoid signaling is involved in osteoarthritis and its modulation might be effective in the management of osteoarthritis [14]. BCP shows analgesic activity [15] and, most interestingly, showed anti-inflammatory and anti-oxidant activity in arthritic rats [16]. However, the exact mechanism of this effect has not been yet fully elucidated.

An interaction between the CB2 receptor and peroxisome proliferator-activated receptor gamma (PPAR-γ) has been shown and suggested as explanation for its curative effects, at least in diet induced neurobehavioral change, dyslipidemia, and vascular inflammation; in particular, BCP showed anxiolytic, anti-oxidant, and anti-inflammatory effects mediated by both PPAR-γ and CB2R [17,18]. Once CB2R is stimulated by BCP, other pathways are activated such as SIRT-1/PGC-1α and AMPK/CREB [19], therefore BCP may exert its anti-inflammatory activity thanks to PPAR-γ activation following CB2R stimulation. Interestingly, PPAR-γ also exerts an important role in rheumatoid arthritis [20,21,22].

However, its involvement in BCP anti-arthritic effects has not yet been investigated. The aim of this study was to better characterize the therapeutic activity of BCP and to get insights into PPAR-γ involvement in the protective effects of BCP in a collagen antibody induced arthritis (CAIA) experimental model.

## 2. Results

### 2.1. BCP Treatment Reduces the Severity of Arthritis

**Figure 1:**
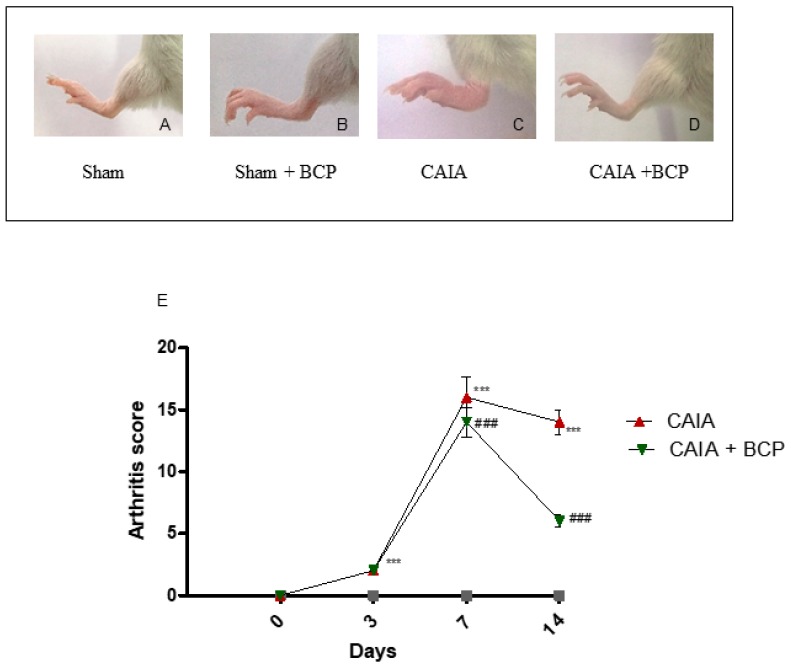
Macroscopic evaluation of arthritis in Sham (**A**), Sham + BCP (**B**), CAIA (**C**), and CAIA + BCP (**D**) mice. Evaluation was performed at the end of the experiment. The graph (**E**) represents arthritis score in Sham, Sham + BCP, CAIA, and CAIA + BCP mice monitored during the experiment. The score obtained from the Sham and Sham + BCP group was 0 at all time points, thus should be assumed as coincident with the *X*-axis of the graph. Data are expressed as means ± SD. *** *p* < 0.0001 vs. Sham and Sham + BCP; ^###^ *p* < 0.0001 vs. CAIA.

The first manifestations of arthritis were erythema and swelling of 1 or more ankle joints. In the CAIA group, 100% of mice developed arthritis at day 7. Oral administration of 10 mg/kg of BCP significantly reduced the development of arthritis compared to CAIA mice that did not receive BCP at day 14 (Figure 1A–E), and no adverse events were observed throughout the experiment. The progression and the severity of the disease were evaluated using an arthritis index. The disease was always progressive. The arthritis score obtained from Sham and Sham + BCP group was 0 at all time points, whereas the score was significantly increased in the CAIA group compared to Sham groups; BCP treatment significantly reduced the arthritis score at the end of the experiment (Figure 1E). However, BCP treated animals showed a higher disease score than sham mice. This suggests that BCP attenuates but does not fully revert the severity of arthritis.

### 2.2. BCP Preserves Articular Cartilage

**Figure 2:**
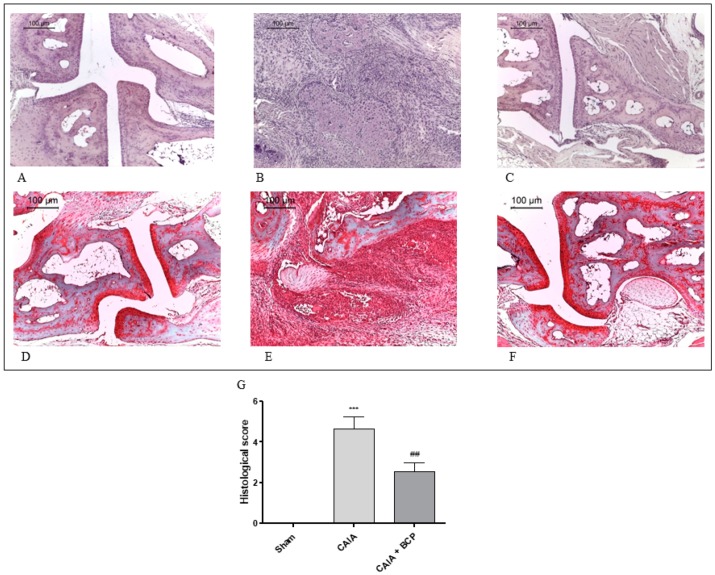
Representative H&E and Safranin O staining of joints from mice: Sham (**A**,**D**), CAIA (**B**,**E**) and CAIA + BCP (**C**,**F**). The graph (**G**) represents the histological score. Data are expressed as means ± SD. *** *p* < 0.0001 vs. Sham; ^##^ *p* < 0.001 vs. CAIA.

Joints from CAIA mice showed severe arthritis with complete ulceration and hyaline cartilage matrix loss exposing the subchondral bone to the intra-articular space. In the adjacent areas, severe cartilage fibrillation and moderate inflammatory granulocytes infiltration extended to the subchondral bone were observed. Chondroid metaplasia of the synovial stroma and synovial hyperplasia were also detected. A fibrovascular granulation tissue filling bone marrow spaces in close proximity to the exposed subchondral bone surface was noted. (Figure 2B). Histological alterations of joints were significantly reduced in BCP-treated mice. In particular, inflammatory infiltrate was decreased and cartilage was preserved (Figure 2C). There were no signs of pathology in Sham mice (Figure 2A) and in the Sham + BCP group (data not shown).

Normal appearance of both extracellular matrix deposition and collagen fiber organization were observed in the joints of Sham mice following Safranin O—Fast Green staining (Figure 2D). Severe arthritis fibrocartilaginous separation with edema, mucoid degeneration, and unorganized collagen fibers were revealed in CAIA joints (Figure 2E). Joints of BCP-treated animals showed lower bands of degenerated extracellular matrix (Figure 2F).

### 2.3. BCP Exerts Anti-Inflammatory Activity by Reducing Pro-Inflammatory Cytokines Levels and Expression

**Figure 3:**
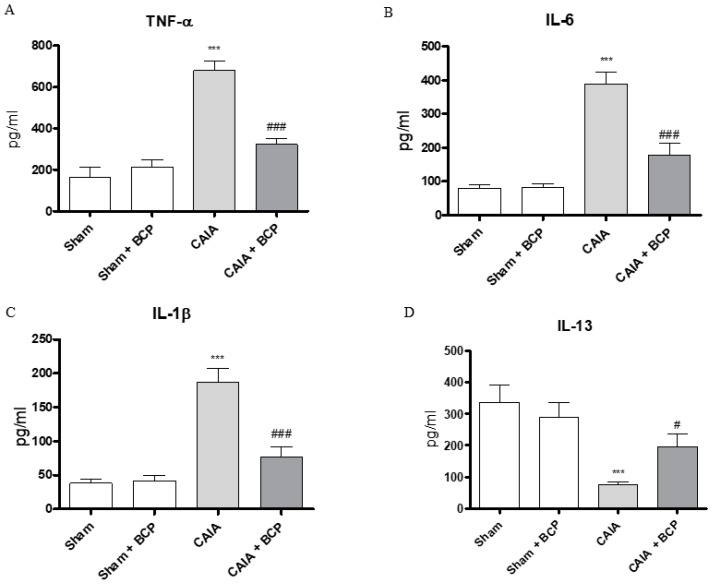
The graphs represent the levels of TNF-α (**A**), IL-6 (**B**), IL-1β (**C**), and IL-13 (**D**) in serum collected from each group at day 15. Levels of cytokines were studied by enzyme linked immunosorbent assay (ELISA). Values shown are expressed as the means ± SD. *** *p* < 0.0001 vs. Sham and Sham + BCP; ^###^ *p* < 0.0001 and ^#^ *p* < 0.05 vs. CAIA.

**Figure 4:**
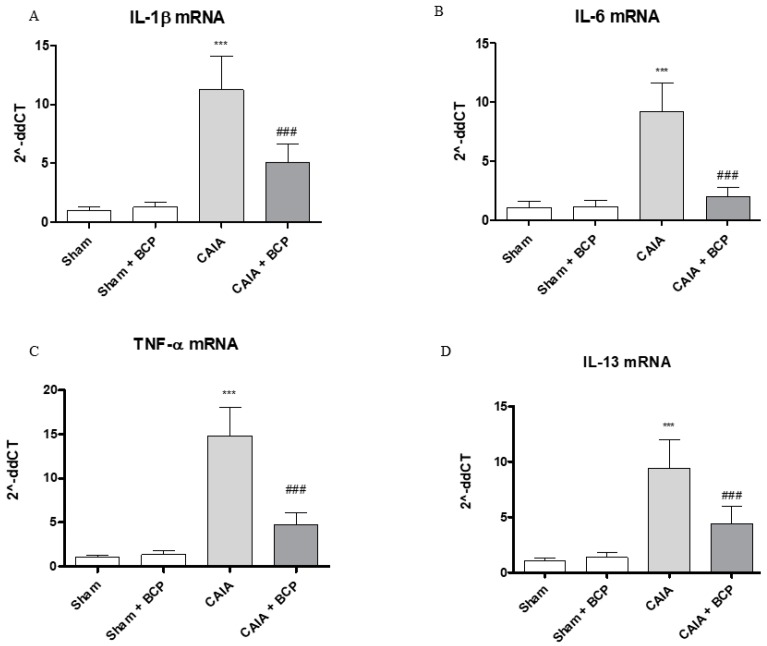
The graphs represent qPCR results of IL-1β (**A**), IL-6 (**B**), TNF-α (**C**), and IL-13 (**D**) mRNA expression from joints. Data are expressed as means ± SD. *** *p* < 0.0001 vs. Sham and Sham + BCP; ^###^ *p* < 0.0001 vs. CAIA.

CAIA mice showed increased circulating levels of TNFα, IL-6, IL-1β and decreased IL-13 levels, as anti-inflammatory cytokine, compared to Sham animals. BCP oral administration caused a significant reduction of all circulating pro-inflammatory cytokines. In contrast, IL-13 was significantly increased following BCP treatment (Figure 3A–D). TNFα, IL-6, and IL-1β were also increased in joints of CAIA animals, as demonstrated by mRNA expression results in Figure 4. mRNA expression of pro-inflammatory cytokines was significantly reduced in BCP-treated mice compared to the CAIA group (Figure 4A–C). IL-13 mRNA expression was decreased in joints of CAIA mice, whereas treatment with BCP significantly increased mRNA expression compared to the CAIA group, confirming the results obtained for circulating IL-13 levels (Figure 4D).

### 2.4. BCP Protects Articular Cartilage by Modulating Metalloproteinases 3 and 9 Expression

**Figure 5:**
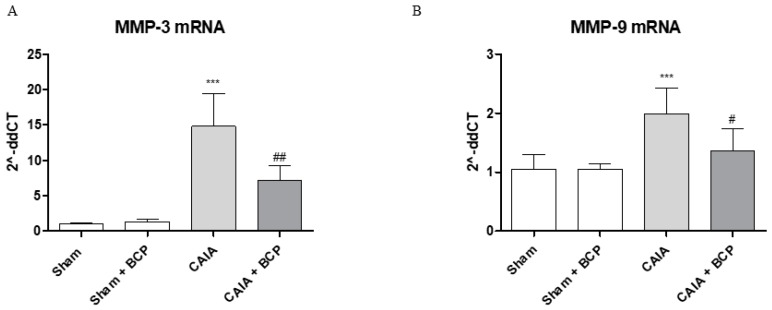
The graphs represent qPCR results of MMP3 (**A**) and MMP-9 (**B**) mRNA expression from joints. Data are expressed as means ± SD. *** *p* < 0.0001 vs. Sham and Sham + BCP; ^##^ *p* < 0.001 and ^#^ *p* < 0.05 vs. CAIA.

The joints of CAIA mice showed an increased mRNA expression of both MMP3 and MMP9 compared to Sham animals, thus confirming their active role in cartilage destruction. BCP treatment modulated MMPs expression, thus significantly reducing MMP3 and MMP9 mRNA expression compared to CAIA animals (Figure 5A,B).

### 2.5. BCP Acts Through NF-ĸB and PPARγ Modulation

**Figure 6:**
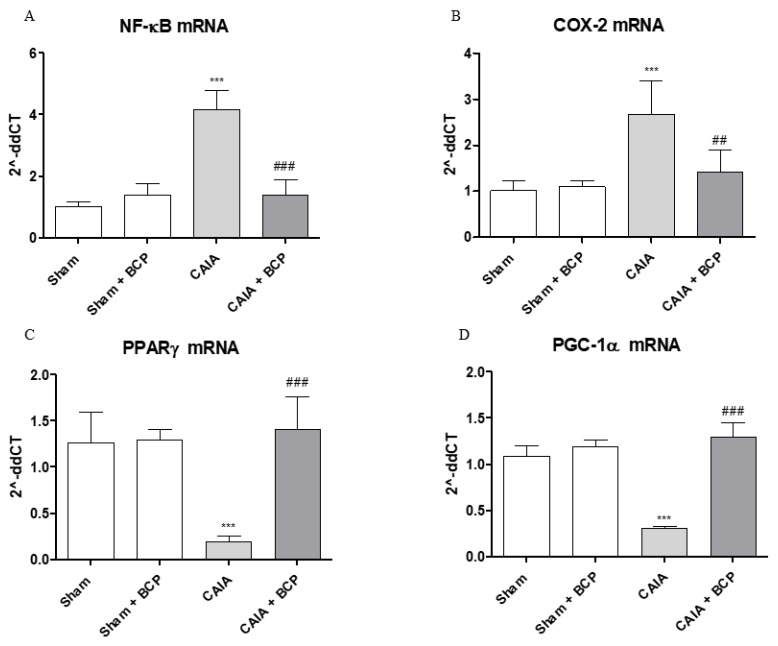
The graphs represent qPCR results of NF-ĸB (**A**), COX-2 (**B**), PPARγ (**C**), and PGC-1α (**D**) mRNA expression from joints. Data are expressed as means ± SD. *** *p* < 0.0001 vs. Sham and Sham + BCP; ^###^ *p* < 0.0001, ^##^ *p* < 0.001 vs. CAIA.

**Figure 7:**
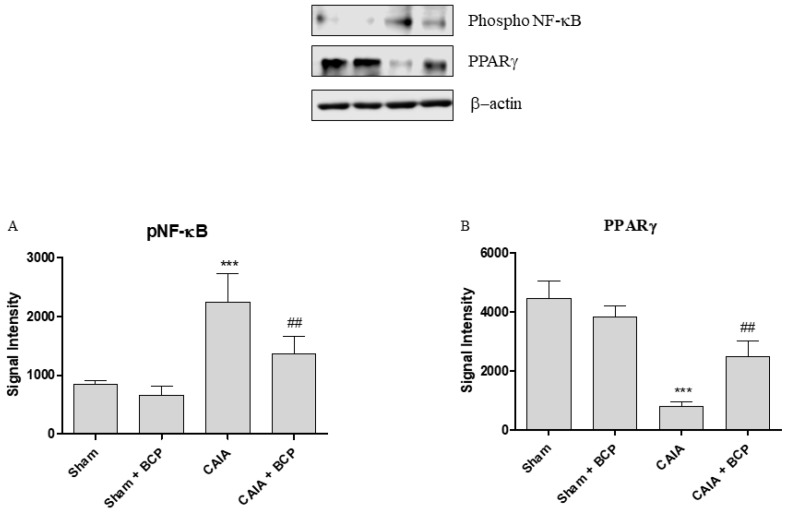
The graphs represent Western blot results of NF-ĸB (**A**) and PPARγ (**B**) protein expression from joints. Data are expressed as means ± SD. *** *p* < 0.0001 vs. Sham and Sham + BCP; ^##^ *p* < 0.001 vs. CAIA.

Arthritis caused an increased expression of NF-ĸB and COX-2 in joints of mice. BCP administration caused a significant reduction of the transcription factor NF-ĸB and COX-2, thus confirming that the anti-inflammatory effect of BCP is also mediated by NF-ĸB and COX-2 inhibition (Figure 6A,B and Figure 7A). Moreover, BCP anti-arthritic and anti-inflammatory effects were elicited through PPAR-γ activation, as shown in Figure 6C and Figure 7B. A significant reduction of PPAR-γ expression and of its co-activator PGC-1α was observed in joints of arthritic mice. BCP treatment caused a significant increase of both PPAR-γ and PGC-1α expression compared to CAIA mice (Figure 6C,D and Figure 7B).

### 2.6. BCP Anti-Arthritic Effect Occurs Through PPAR-γ and PGC-1α Activation

**Figure 8:**
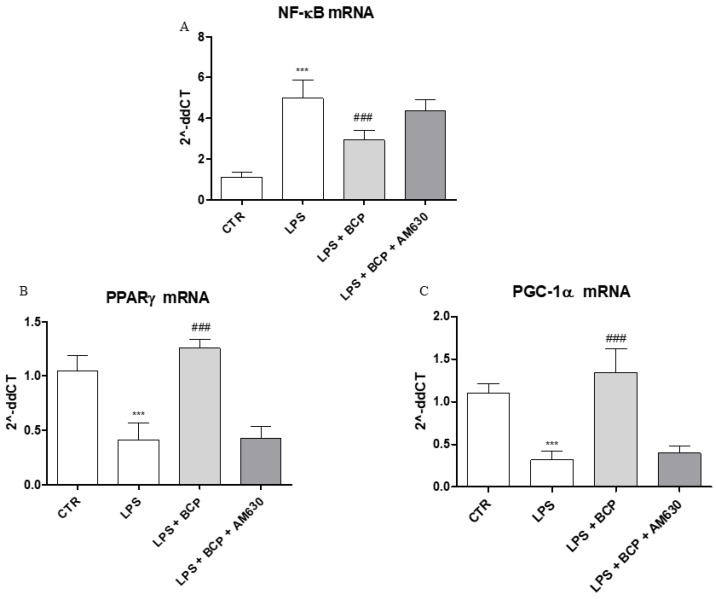
The graphs show qPCR results of (**A**) NF-ĸB, (**B**) PPARγ, and (**C**) PGC-1α mRNA expression from human chondrocytes. Data are expressed as means ± SD. *** *p* < 0.0001 vs. Control (CTR); ^###^ *p* < 0.0001 vs. LPS.

An in vitro experiment on human chondrocytes was performed to demonstrate BCP mechanism of action. This experimental paradigm confirmed in vivo results and showed that BCP caused a marked reduction of NF-ĸB mRNA expression compared to LPS-stimulated chondrocytes (Figure 8A), once again demonstrating a BCP anti-inflammatory effect. In addition, BCP caused a significant increase of PPAR-γ and PGC-1α mRNA expression compared to cells incubated with LPS alone (Figure 8B,C). AM630, a CB2 receptor antagonist, abrogated BCP effects in all targets (Figure 8A–C).

## 3. Discussion

Chronic inflammation together with erosion and destruction of joints characterize rheumatoid arthritis. Nowadays, the available therapeutic approaches allow satisfactory management of RA with patients achieving remission or having minimal disease activity. However, a huge amount of RA patients do not respond to these different and often expensive classes of anti-rheumatic agents and, in addition, they complain of severe adverse effects [23]. Therefore, it is not surprising that the interest of scientists has been caught by complementary and different therapeutic strategies, specifically compounds of natural origin and nutraceuticals, which have gained interest in the management of RA, especially in elderly patients [24,25,26]. In fact, a growing number of patients seek remedy in natural compounds, because they believe that they are effective and safe [27,28].

BCP is a natural compound that has been shown to act as an analgesic, antioxidant, anti-microbial, anti-inflammatory, and antifungal agent [19]. BCP binds the type 2 cannabinoid receptor which is associated with an intracellular signaling cascade triggered by G-protein activation [29]. CB2 receptor expression has been demonstrated in cells of the peripheral immune system, and this sub-type of cannabinoid receptor has been linked to a modulation of the inflammatory and immune reaction. More recent investigation has pointed out the presence of CB2 receptors in discrete central nervous system regions [29]. In agreement with these findings, a neuroprotective effect of BCP has also been reported [11].

As far as the anti-arthritic effects of BCP are concerned, a previous study has suggested that this phytocannabinoid tempers the inflammatory cascade in arthritic rats, but the precise mechanism of action underlying this effect has not been fully elucidated [16]. Indeed, this previous report was primarily aimed at assessing the effects of BCP treatment on systemic inflammation and oxidative stress and to evaluate the hepatic safety of the treatment [16]. More specifically, the study did not deeply analyze the “clinical” benefits of the phytocannabinoid and did not investigate the several inflammatory endpoints, including pro-inflammatory cytokines and metalloproteinases. Our study, for the first time, clearly shows that BCP ameliorates the clinical signs of arthritis. This beneficial effect was associated with a marked reduction in several inflammatory biomarkers and with a clear-cut reduction in histological joint damage. Indeed, the clinical symptoms of arthritis are mainly due to a plethora of lipid and protein inflammatory mediators that are produced in response to immune reaction boosting, whereas the available treatments dampen the circulating levels of these circulating inflammatory markers. Therefore, it is required that the newly proposed therapeutic approach has to be accurately evaluated in appropriate and pertinent animal models.

Indeed, from a translational point view, the use of an experimental model characterized by reproducible and clinically relevant biomarkers and readouts that allow a close monitoring and follow-up of the disease progression is of paramount importance. In the present study, a collagen antibody induced arthritis (CAIA) experimental model was used in mice. This experimental paradigm efficiently reflects the human pathology and possesses a high translational potential [30]. In fact, this model offers a valuable scenario for evaluating a treatment that can be proposed as an appropriate candidate for the management of rheumatoid arthritis.

BCP administration significantly ameliorated the signs and the clinical symptoms associated with the development of arthritis. The severity of the disease, analyzed by the means of the clinical and histological scores, was markedly reduced by the treatment with the natural CB2 agonist. This finding represents the first clear-cut experimental evidence in support of an anti-arthritic effect of BCP.

A vast array of pro-inflammatory factors is produced either locally in the affected joints and then released in the bloodstream by the primed inflammatory cells; furthermore, they may be used as clinically relevant biomarkers. Interestingly, the cannabinoid receptor agonist reduced pro-inflammatory cytokines such as TNF-α, IL-6, Il-1β and increased the anti-inflammatory cytokine IL-13. BCP also suppressed joint expression of TNF-α, IL-6, Il-1β and of MMP 3 and MMP9.

COX2 and NF-ĸB expression was studied to characterize the precise mechanism underlying the curative effect of BCP in RA. Joints of arthritic animals administered with vehicle showed an enhanced expression of COX2 and NF-ĸB. BCP administration dampened the augmented activity of both inflammatory molecules. This effect is the consequence of the intracellular signaling cascade initiated by the BCP-induced triggering of the CB2 receptors, which leads to a blunting of NF-ĸB activation.

However, the hypothesis that BCP-induced NF-ĸB inhibition might exert beneficial effects in arthritis by negatively modulating other inflammatory signaling, such as Wnt5a [31], cannot at this moment ruled out

Interestingly, arthritic joints also had a significantly reduced expression of the peroxisome proliferator-activator receptor γ coactivator-1α (PGC-1α) and PPAR-γ compared to sham animals. All these changes induced by the induction of arthritis were cured by BCP treatment.

In recent years, it has become evident that endocannabinoids trigger the activation of a specific nuclear receptor family, the PPARs (peroxisome proliferator-activated receptors) [32,33]. These receptors are classically divided into α, β, δ, and γ, and they are involved in cellular homeostasis and differentiation as well as in the regulation of lipid and glucose metabolism. Furthermore, PPAR-γ modulates inflammation and ischemia and may be pharmacologically activated to exert anti-inflammatory and anti-ischemic effects. PPAR activation by cannabinoid agonists may occur via two distinct mechanisms: a direct engagement of the nuclear receptor or alternatively a cannabinoid receptor boosting of intracellular events leading to PPAR stimulation (the so-called ligand independent activation). The first hypothesis has been ruled out, while the second one has been suggested by several experimental findings [33]. In this last scenario, PGC-1α plays a key role: in fact, following its expression, it engages and triggers PPAR-γ acting in a ligand-independent manner.

As previous underlined, CAIA-induced arthritis caused a downregulation of both PPAR-γ and its main regulatory coactivator PGC-1α in arthritic joints. It may be speculated that this phenomenon concurs to amplify inflammation, creating a negative loop. BCP reverted the impairment in the ligand-independent PPAR-γ signaling, leading us to hypothesize that this mechanism may explain, at least in part, the anti-arthritic effect of BCP. In addition, an in vitro experiment was performed to confirm this mechanistic hypothesis using human articular chondrocytes stimulated with LPS. BCP decreased the enhanced NF-ĸB activation and increased the reduced expression of PGC-1α and PPAR-γ. These effects were abolished by AM630, an antagonist of the CB2 receptor, while bisphenol-A-dyglycidyl ether (BADGE) did not modify PGC-1α expression (unpublished data). Therefore, in agreement with previous studies [17,18], an involvement of both CB2 and PPAR-γ was demonstrated in the anti-arthritic effect of BCP, and the effect induced by the phytocannabinoid on the peroxisome proliferator-activator receptor γ coactivator-1α (PGC-1α) is entirely dependent by the CB2 receptor.

In conclusion, the obtained results suggest that BCP ameliorates arthritis through a cross-talk between CB2 and PPAR-γ. In addition, this study underlines the translational potential of BCP that deserves to be deeply investigated in randomized clinical trials.

## 4. Materials and Methods

### 4.1. CAIA Induction and Treatment

Forty balb/c mice (25–30 g; Charles River, Calco, Italy) were housed in the Animal Facility of the Department of Clinical and Experimental Medicine under controlled environmental conditions (12 h light–dark cycle, 24 °C). Mice were provided with standard food and water ad libitum and all the experiments were performed in compliance with the standards for care and use of animals as stated in the Directive 2010/63/EU, and the ARRIVE guidelines [34]; all experimental procedures were evaluated and approved by the Ethics Committee of the University of Messina and by the Italian Ministry of Health (#756/2016-PR).

Collagen type II antibody-induced arthritis (CAIA) was induced in 20 balb/c mice as follows: on day 0, animals were intraperitoneally (ip) injected with 1.5 mg of 5-clone monoclonal antibody cocktail (Chondrex); on day 3, mice received LPS (50 μg/100 μL/ip) [35]. At day 3, after the completion of the protocol induction, CAIA animals were daily treated by oral gavage with BCP (10 mg/kg/100 μL, CAIA + BCP) or its vehicle (100 μL of corn oil) until the end of the experiment (14th day). The dose was chosen in agreement with previous pilot experiments in our laboratory (data not shown). Sham animals (*n* = 20) received 100 μL of corn oil (*n* = 10, Sham) or BCP (*n* = 10, Sham + BCP). Animals were killed on day 15 and both blood and hind limbs were collected for further analysis.

### 4.2. Evaluation of Arthritis

Evaluation of arthritis was performed throughout the study. Development of arthritis was monitored at the beginning of the experiment (after the antibody cocktail injection), at day 3 (following LPS injection), at day 7, and at the end of the experiment to appreciate alterations throughout the study. The severity of arthritis was blindly evaluated in each limb with a scale of 0–4, where 0 = no macroscopic signs of arthritis, 1 = swelling of 1 group of joints (i.e., wrist or ankle joints), 2 = 2 groups of swollen joints, 3 = 3 groups of swollen joints, and 4 = swelling of the entire limb. The maximum score for each animal was 16.

### 4.3. Cell Cultures and Treatments

Human articular chondrocytes (ScienCell, CA, USA) were cultured in a chondrocyte medium (Cat. #4651, ScienCell, CA, USA) in addition to antibiotic mixture (1%) and incubated at 37 °C with 5% CO_2_. Chondrocytes were cultured in six well culture plates at a density of 2.5 × 10^5^ cells/well and were stimulated with LPS (2 μg/mL; Escherichia coli serotype 055:B5; Sigma-Aldrich, Saint Louis, MO, USA) alone or in combination with BCP (Sanherb Biotech inc., China) at the dose of 10 μg/mL. Moreover, a group of LPS-stimulated chondrocytes were treated with both BCP and AM630, a CB2 receptor antagonist, at the dose of 100 nM (Tocris Bioscience); AM630 was added 2 hours before BCP treatment. Cells were collected following 4 h of incubation with all treatments.

### 4.4. Histological Analysis

Joints were removed from the hind limbs of all groups of animals at the end of the experiment and fixed in 10% formalin. Joints were decalcified in 10% EDTA for 2–3 weeks and embedded in paraffin. Paraffin-embedded sections were cut and stained with hematoxylin and eosin and with Safranin O for histologic assessment. Arthritis severity in histologic samples was determined by cumulative assessment of synovial inflammation. Samples were scored for synovial inflammation on a scale of 0–5: 0 = normal, 1 = minimal, 2 = mild, 3 = moderate, 4 = marked, and 5 = severe.

### 4.5. Quantification of Pro- and Anti-Inflammatory Cytokines by Enzyme-Linked Immunosorbent Assay (ELISA)

TNF-α, IL-6, IL-1β, and IL-13 levels were evaluated in the serum of the animals at the end of the experiment. All cytokines were measured using ELISA kits (Invitrogen, Carlsbad, CA, USA) according to the recommendations of the manufacturer. Samples were run in duplicate and the obtained results were interpolated with the respective standard curves. For each sample, the mean of the duplicates was used and expressed in pg/mL.

### 4.6. Real Time (RT) PCR Assay

Total RNA was extracted from the joints of mice and from cultured chondrocytes at the end of the experimental procedures using Trizol LS reagent (Invitrogen, Carlsbad, CA, USA). 2 μg of RNA was reverse transcribed in a final volume of 20 μL using a Superscript VILO kit (Invitrogen), following RNA quantification with a spectrophotometer (NanoDrop Lite, Thermo Fisher). cDNA (1 μL) was added to the EvaGreen qPCR Master Mix (Biotium Inc., Fremont, CA, USA) (20 μL per well). Samples were run in duplicate and GADPH was used as the housekeeping gene; the reaction was performed using the 2-step thermal protocol recommended by the manufacturer. The final primer concentration selected to perform the analysis was 10 μM. Target genes were IL-1β, IL-6, TNF-α, IL-13, MMP-3, MMP-9, NF-ĸB, COX-2, PPARγ, and PGC-1α.

Primers used for targets and reference genes are listed below:

GADPH

Fw:5′GTCAAGGCTGAGAATGGGAA3′

Rv:5′ATACTCAGCACCAGCATCAC3′;

IL-1β

Fw:5′GCCCATCCTCTGTGACTCAT3′

Rv:5′AGGCCACAGGTATTTTGTCG3′;

IL-6

Fw:5′AGTTGCCTTCTTGGGACTGA3′

Rv:5′TCCACGATTTCCCAGAGAAC3′;

TNF-α

Fw:5′AGCCCCCAGTCTGTATCCTT3′

Rv:5′CTCCCTTTGCAGAACTCAGG3′;

IL-13

Fw:5′CAGCTCCCTGGTTCTCTCAC3′

Rv:5′CCACACTCCATACCATGCTG′;

MMP-3

Fw:5′CAGACTTGTCCCGTTTCCAT 3′

Rv:5′GGTGCTGACTGCATCAAAGA3′;

MMP-9

Fw:5′CGTCGTGATCCCCACTTACT3′

Rv:5′AACACACAGGGTTTGCCTTC3′;

NF-ĸB

Fw:5′GCCAGAAGAGGGTGTCAGAG3′

Rv:5′ TCGAAATCCCCTCTGTTTTG ′;

COX-2

Fw:5′CCCCCACAGTCAAAGACACT3′

Rv:5′CTCATCACCCCACTCAGGAT3′;

PPARγ

Fw:5′CCAACTTCGGAATCAGCTCT 3′

Rv:5′CAACCATTGGGTCAGCTCTT3′;

PGC-1α

Fw:5′ATGTGTCGCCTTCTTGCTCT 3′

Rv:5′ATCTACTGCCTGGGGACCTT3′;

Primers used for targets and reference genes for samples collected from human cells are listed below:

NF-ĸB

Fw:5′CTGGAAGCACGAATGACAGA3′

Rv:5′ TGAGGTCCATCTCCTTGGTC3′;

PPARγ

Fw:5′ GGAGCAAACGACACCAGATT3′

Rv:5′ TCAAAGGAGTGGGAGTGGTC3′;

PGC-1α

Fw:5′AGGCAGAAGGCAATTGAAGA3′

Rv:5′ TTTCAAGAGCAGCAAAAGCA3′;

Results were calculated using the 2^−ΔΔCt^ method and expressed as n-fold increase in gene expression using the Sham group as calibrator.

### 4.7. Western Blot

Protein extraction was performed in joints for Western Blot analysis, as previously described [36]. About 30 µg of proteins were loaded and specific antibodies were used to evaluate phospho NF-ĸB (Cell Signaling, #3033, Beverly, MA, USA) and PPARγ (Cell Signaling, #2443, Beverly, MA, USA); β-actin (Cell Signaling, #4967, Beverly, MA, USA) was used as loading control. The images were obtained using specific software (DiGit Blot Scanner with Image Studio 4.0 software, LI-Cor, Lincoln, NE, USA), and densitometric data were expressed as integrated intensity.

### 4.8. Statistical Analysis

All data are expressed as means ± S.D. Different groups were compared and analyzed using one-way ANOVA with Tukey post-test for intergroup comparisons. *p* values less than 0.05 were considered significant. Graphs were drawn using GraphPad Prism (version 5.0 for Windows).

## Abbreviations

β-caryophyllene, BCP; collagen antibody induced arthritis, CAIA; cannabinoid, CB; peroxisome proliferator-activated receptor gamma, PPAR-γ, Nuclear Factor kappa B, NFĸB; PPARγ coactivator-1 alpha, PGC-1α; Tumor Necrosis Factor alpha, TNF-α; interleukin, IL; metalloproteinase, MMP; lipopolysaccaride, LPS.

## References

[1] Scott D.L., Wolfe F., Huizinga T.W. (2010). Rheumatoid arthritis. Lancet.

[2] Smolen J.S., Aletaha D., Barton A., Burmester G.R., Emery P., Firestein G.S., Kavanaugh A., McInnes I.B., Solomon D.H., Strand V. (2018). Rheumatoid arthritis. Nat. Rev. Dis. Primers.

[3] Zamanpoor M. (2018). The genetic pathogenesis, diagnosis and therapeutic insight of rheumatoid arthritis. Clin. Genet..

[4] Yu H.C., Lu M.C. (2019). The roles of anti-citrullinated protein antibodies in the immunopathogenesis of rheumatoid arthritis. Ci Ji Yi Xue Za Zhi.

[5] Chen Z., Bozec A., Ramming A., Schett G. (2019). Anti-inflammatory and immune-regulatory cytokines in rheumatoid arthritis. Nat. Rev. Rheumatol..

[6] Itoh Y. (2017). Metalloproteinases in Rheumatoid Arthritis: Potential Therapeutic Targets to Improve Current Therapies. Prog. Mol. Biol. Transl. Sci..

[7] Hazlewood G.S., Barnabe C., Tomlinson G., Marshall D., Devoe D.J., Bombardier C. (2016). Methotrexate monotherapy and methotrexate combination therapy with traditional and biologic disease modifying anti-rheumatic drugs for rheumatoid arthritis: A network meta-analysis. Cochrane Database Syst. Rev..

[8] Hazlewood G.S., Bombardier C., Tomlinson G., Thorne C., Bykerk V.P., Thompson A., Tin D., Marshall D.A. (2016). Treatment preferences of patients with early rheumatoid arthritis: A discrete-choice experiment. Rheumatology.

[9] Choi M.Y., Barnabe C., Barber C.E., Bykerk V., Pope J.E., Hazlewood G.S. (2018). Randomized controlled trials of biologic treatment with methotrexate in RA may not reflect real world practice: A systematic review and assessment of pragmaticism. Arthritis Care Res. (Hoboken).

[10] Donges E., Staatz C.E., Benham H., Kubler P., Hollingworth S.A. (2017). Patterns in use and costs of conventional and biologic disease-modifying anti-rheumatic drugs in Australia. Clin. Exp. Rheumatol..

[11] Machado K.D.C., Islam M.T., Ali E.S., Rouf R., Uddin S.J., Dev S., Shilpi J.A., Shill M.C., Reza H.M., Das A.K. (2018). A systematic review on the neuroprotective perspectives of beta-caryophyllene. Phytother. Res..

[12] Russo E.B. (2016). Beyond Cannabis: Plants and the Endocannabinoid System. Trends Pharmacol. Sci..

[13] Gong J.P., Onaivi E.S., Ishiguro H., Liu Q.R., Tagliaferro P.A., Brusco A., Uhl G.R. (2006). Cannabinoid CB2 receptors: Immunohistochemical localization in rat brain. Brain Res..

[14] La Porta C., Bura S.A., Llorente-Onaindia J., Pastor A., Navarrete F., García-Gutiérrez M.S., De la Torre R., Manzanares J., Monfort J., Maldonado R. (2015). Role of the endocannabinoid system in the emotional manifestations of osteoarthritis pain. Pain.

[15] Fidyt K., Fiedorowicz A., Strządała L., Szumny A. (2016). β-caryophyllene and β-caryophyllene oxide-natural compounds of anticancer and analgesic properties. Cancer Med..

[16] Ames-Sibin A.P., Barizão C.L., Castro-Ghizoni C.V., Silva F.M.S., Sá-Nakanishi A.B., Bracht L., Bersani-Amado C.A., Marçal-Natali M.R., Bracht A., Comar J.F. (2018). β-Caryophyllene, the major constituent of copaiba oil, reduces systemic inflammation and oxidative stress in arthritic rats. J. Cell. Biochem..

[17] Youssef D.A., El-Fayoumi H.M., Mahmoud M.F. (2019). Beta-caryophyllene alleviates diet-induced neurobehavioral changes in rats: The role of CB2 and PPAR-γ receptors. Biomed. Pharmacother..

[18] Youssef D.A., El-Fayoumi H.M., Mahmoud M.F. (2019). Beta-caryophyllene protects against diet-induced dyslipidemia and vascular inflammation in rats: Involvement of CB2 and PPAR-γ receptors. Chem. Biol. Interact..

[19] Sharma C., Al Kaabi J.M., Nurulain S.M., Goyal S.N., Kamal M.A., Ojha S. (2016). Polypharmacological Properties and Therapeutic Potential of β-Caryophyllene: A Dietary Phytocannabinoid of Pharmaceutical Promise. Curr. Pharm. Des..

[20] Kwon E.J., Park E.J., Choi S., Kim S.R., Cho M., Kim J. (2017). PPARγ agonist rosiglitazone inhibits migration and invasion by downregulating Cyr61 in rheumatoid arthritis fibroblast-like synoviocytes. Int. J. Rheum. Dis..

[21] Vasheghani F., Zhang Y., Li Y.H., Blati M., Fahmi H., Lussier B., Roughley P., Lagares D., Endisha H., Saffar B. (2015). PPARγ deficiency results in severe, accelerated osteoarthritis associated with aberrant mTOR signalling in the articular cartilage. Ann. Rheum. Dis..

[22] Wang R.C., Jiang D.M. (2014). PPAR-γ agonist pioglitazone affects rat gouty arthritis by regulating cytokines. Genet. Mol. Res..

[23] Favalli E.G., Raimondo M.G., Becciolini A., Crotti C., Biggioggero M., Caporali R. (2017). The management of first-line biologic therapy failures in rheumatoid arthritis: Current practice and future perspectives. Autoimmun. Rev..

[24] Dudics S., Langan D., Meka R.R., Venkatesha S.H., Berman B.M., Che C.T., Moudgil K.D. (2018). Natural Products for the Treatment of Autoimmune Arthritis: Their Mechanisms of Action, Targeted Delivery, and Interplay with the Host Microbiome. Int. J. Mol. Sci..

[25] Albini A., Bassani B., Baci D., Dallaglio K., Gallazzi M., Corradino P., Bruno A., Noonan D.M. (2019). Nutraceuticals and “repurposed” drugs of phytochemical origin in prevention and interception of chronic degenerative disease and cancer. Curr. Med. Chem..

[26] Zhao S., Otieno F., Akpan A., Moots R.J. (2017). Complementary and Alternative Medicine Use in Rheumatoid Arthritis: Considerations for the Pharmacological Management of Elderly Patients. Drugs Aging.

[27] DeSalvo J.C., Skiba M.B., Howe C.L., Haiber K.E., Funk J.L. (2018). Natural Product Dietary Supplement Use by Individuals with Rheumatoid Arthritis: A Scoping Review. Arthritis Care Res. (Hoboken).

[28] Hall J.J., Dissanayake T.D., Lau D., Katz S.J. (2017). Self-reported use of natural health products among rheumatology patients: A cross-sectional survey. Musculoskelet. Care.

[29] Lucas C.J., Galettis P., Schneider J. (2018). The pharmacokinetics and the pharmacodynamics of cannabinoids. Br. J. Clin. Pharmacol..

[30] Gillooly K.M., Pulicicchio C., Pattoli M.A., Cheng L., Skala S., Heimrich E.M., McIntyre K.W., Taylor T.L., Kukral D.W., Dudhgaonkar S. (2017). Bruton’s tyrosine kinase inhibitor BMS-986142 in experimental models of rheumatoid arthritis enhances efficacy of agents representing clinical standard-of-care. PLoS ONE.

[31] Ge X.P., Gan Y.H., Zhang C.G., Zhou C.Y., Ma K.T., Meng J.H., Ma X.C. (2011). Requirement of the NF-κB pathway for induction of Wnt-5A by interleukin-1β in condylar chondrocytes of the temporomandibular joint: Functional crosstalk between the Wnt-5A and NF-κB signaling pathways. Osteoarthr. Cartil..

[32] O’Sullivan S.E. (2007). Cannabinoids go nuclear: Evidence for activation of peroxisome proliferator-activated receptors. Br. J. Pharmacol..

[33] Pistis M., O’Sullivan S.E. (2017). The Role of Nuclear Hormone Receptors in Cannabinoid Function. Adv. Pharmacol..

[34] Kilkenny C., Browne W.J., Cuthill I.C., Emerson M., Altman D.G. (2010). Improving Bioscience Research Reporting: The ARRIVE Guidelines for Reporting Animal Research. PLoS Biol..

[35] Hutamekalin P., Saito T., Yamaki K., Mizutani N., Brand D.D., Waritani T., Terato K., Yoshino S. (2009). Collagen antibody-induced arthritis in mice: Development of a new arthritogenic 5-clone cocktail of monoclonal anti-type II collagen antibodies. J. Immunol. Methods.

[36] Marini H., Polito F., Altavilla D., Irrera N., Minutoli L., Calò M., Adamo E.B., Vaccaro M., Squadrito F., Bitto A. (2010). Genistein aglycone improves skin repair in an incisional model of wound healing: A comparison with raloxifene and oestradiol in ovariectomized rats. Br. J. Pharmacol..

